# Sox6 suppression induces RA-dependent apoptosis mediated by BMP-4 expression during neuronal differentiation in P19 cells

**DOI:** 10.1007/s11010-015-2607-8

**Published:** 2015-11-20

**Authors:** Michiko Hamada-Kanazawa, Daisuke Ogawa, Masaoki Takano, Masaharu Miyake

**Affiliations:** Division of Biopharmaceutical Sciences, Faculty of Pharmaceutical Sciences, Kobe-Gakuin University, Minatojima, Chuo-Ku, Kobe, 650-8586 Japan

**Keywords:** Sox6, P19, BMP-4, Retinoic acid, Apoptosis, BMPRIB

## Abstract

Sox6 is a transcription factor that induces neuronal differentiation in P19 cells; its suppression not only inhibits neuronal differentiation but also induces retinoic acid (RA)-dependent apoptosis of P19 cells. In the present study, we found that Sox6 suppression-induced apoptosis was mediated by activation of caspase 9 and 3. Moreover, we noted a weak leakage of cytochrome c into the cytoplasm from the mitochondria, indicating that apoptosis occurs through a mitochondrial pathway in Sox6-suppressed P19 (P19[anti-Sox6]) cells. Sox6 suppression in the presence of RA also induced the expression and secretion of bone morphogenetic protein 4 (BMP-4). Addition of an anti-BMP-4 antibody for neutralization increased cell viability and led to RA-dependent death of P19[anti-Sox6] cells. Our results indicate that Sox6 suppression induces RA-dependent cell death of P19 cells, mediated by BMP-4 expression and secretion. Normally, high Sox6 expression leads to RA-mediated neuronal differentiation in P19 cells; however, Sox6 deficiency induces production and secretion of BMP-4, which mediates selective cell death. Our findings suggest that Sox6 contributes to cell survival by suppressing BMP-4 transcription during neuronal differentiation.

## Introduction

Sox6, which was initially reported to be a member of the Sox gene family (Sry-type HMG box), functions as a sex-determining SRY/Sry-related factor in humans and mice [1]. Sox transcription factors express their respective specific patterns in the central nervous system during embryogenesis and have also been suggested to be involved in the development of the nervous system [2]. Furthermore, Sox proteins are known to be important factors in neurogenesis [3], and Sox2 and Sox3 are mainly expressed in the developing central nervous system, whereas Sox6 plays a role in the early stages of nervous system development, as it is specifically expressed during the initial stage (post-coitus days 9.5–12.5) of embryogenesis [1]. Sox6 was initially isolated from an adult mouse testis cDNA library [1, 4], after which we isolated cDNA encoding a rat homolog of the previously characterized mouse Sox6 [5].

Sox6 also plays a role in the differentiation of various tissues. A Sox6 null mutant was found in mice, and the results of that study showed that Sox6 protein is involved in maintaining the normal physiological functions of muscle tissues, such as those of the heart [6]. It was also reported that Sox6 is expressed in the testis, skeletal muscle, and cardiac muscle tissues of adult mice, except those involved in the development of the central nervous system [7, 8]. Furthermore, Cohen-Barak et al. found that Sox6-deficient mice had cardiac abnormalities and coronary artery formation [8]. In addition, Sox6 and Sox9 play important roles in bone [9] and cartilage [10] formation.

Our studies have shown that Sox6 plays an important role in the development of the central nervous system. To our knowledge, we were the first to report that Sox6 promotes neuronal differentiation of embryonic carcinoma P19 cells, as its expression in these cells remarkably increased during the induction phase mediated by retinoic acid (RA) and then disappeared at the beginning of neuronal differentiation, which was mediated by withdrawal of RA [11]. Furthermore, Sox6 overexpression without RA caused neuronal differentiation of P19 cells, indicating that Sox6 plays a role in whether P19 cells undergo differentiation to neuronal cells or not. Recently, Sox6 was shown to selectively localize in the substantia nigra pars compacta (SNc) neurons, while Sox6 ablation led to decreased expression of SNc markers and dopamine levels, leading to the conclusion that Sox6 controls neuronal progenitor cells during neocortical development [12]. It was also reported that Sox6 is involved in the differentiation of neocortex interneurons [13] and that Stat3 directly activates the transcription of Sox6 [14].

We also previously reported that suppression of Sox6 in P19 cells led to failure of neuronal differentiation by RA and induced RA-dependent apoptosis [15]. Therefore, it is considered that Sox6 is an essential factor for neuronal differentiation and a determinant of cell survival in RA-exposed neuronal progenitor cells. It is known that redundant neuronal progenitor cells are removed by apoptosis during neurogenesis. Thus, Sox6 may also play an important role in the early stages of neuronal differentiation or during apoptosis, because its suppression led to RA-dependent apoptosis.

In the present study, we found that RA-dependent apoptosis occurred through a mitochondrial pathway in Sox6-suppressed P19 (P19[anti-Sox6]) cells. In addition, bone morphogenetic protein-4 (BMP-4) expression increased at both the mRNA and protein levels, and it was secreted into the medium of cultured P19[anti-Sox6] cells in the presence of RA. Addition of the anti-BMP-4 antibody partially blocked RA-dependent apoptosis, while that of BMP-4 protein induced apoptosis in P19[anti-Sox6] cells in the absence of RA. Our results suggest that Sox6 mediates cell survival by downregulating BMP-4 expression.

## Materials and methods

### Cell line

A cytomegalovirus (CMV) promoter-driven expression vector for anti-Sox6 was constructed by inserting a reversed 1-kb Sox6 fragment into the mammalian expression vector pcDNA3.1/myc/his (Invitrogen). After G418 selection for 2 weeks, 10 clones were isolated by limiting dilution. Stable anti-Sox6 mRNA expression was found to block RA-mediated endogenous Sox6 expression.

### Cell culture

A P19 EC cell line was purchased from American Type Culture Collection, and then maintained in alpha-modified minimum essential medium supplemented with 2.5 % fetal bovine serum (FBS) and 7.5 % calf serum (CS). For induction of neuronal differentiation, the cells were cultured in bacterial-grade dishes (IWAKI) in the presence of 500 nM RA.

### Cell counting

P19 cells were cultured in tissue culture dishes in the presence of RA for 24 or 48 h, and then treated with trypsin for collection. Thereafter, they were resuspended in sheath solution and counted using a Coulter counter.

### Fluorescent staining

P19 cells were cultured in 24-well plates as floating cultures in the presence of RA for 24 or 48 h, then exposed to 1 ng/ml Hoechst 33342 for 30 min at room temperature to determine living nuclear cells or 2 ng/ml of propidium iodide (PI) to determine dead nuclear cells.

### MTT assay

P19 cells were cultured in 96-well plates as floating cultures in the presence of RA for 24 or 48 h. Cell viability was determined with a Cell Proliferation Kit I (Roche) based on the cleavage of the tetrazolium salt MTT by the integrity of mitochondrial functions according to the manufacturer’s instructions.

### Assay of caspase activity

Approximately 10^6^ cells were cultured in bacterial-grade dishes in the presence or absence of RA for 36 h. After incubation, the cells were washed twice with PBS and lysed in PBS containing 0.2 % Triton X-100 on ice for 10 min. After centrifugation at 10,000×*g* for 20 min, Ac-DEVD-pNA cleavage activity in the cell extracts was determined using a Caspase 3 Colorimetric Activity Assay Kit (Chemicon) according to the manufacturer’s instructions. In similar assays, Ac-IETD-pNA was used as a substrate for caspase 8 and Ac-LEHD-pNA as a substrate for caspase 9. To inhibit caspase activity, the cells were incubated with the caspase inhibitor Ac-DEVD-CHO (Calbiochem^®^) for caspase 3, Z-IETD-FMK for caspase 8, and LEHD-CHO for caspase 9 during RA induction.

### Measurement of the expression of cytoplasmic cytochrome c and Bcl family proteins

P19 cells were cultured in bacterial-grade dishes in the presence or absence of RA for 24 h. Then, the cells were collected and washed with PBS, resuspended in 0.34 M sucrose solution (0.34 M sucrose, 20 mM Tris–HCl [pH 7.4], 10 mM KCl, 1.5 mM MgCl_2_, 1 mM EDTA, 1 mM EGTA, 1 mM DTT, Proteinase Inhibitor Cocktail [III]). Cell suspensions were homogenized using a Teflon homogenizer on ice, and then the homogenates were centrifuged at 700×*g* for 10 min at 4 °C to remove the nuclear fraction, after which the supernatants were centrifuged at 1000×*g* for 30 min at 4 °C. The supernatants were used as the cytosol fraction, and the pellets were dissolved with TBS containing 0.5 % Triton X-100 as the mitochondrial fraction. The amount of cytoplasmic cytochrome c in these fractions was measured by sandwich ELISA-based method using a Cytochrome c Mouse/Rat ELISA Quantikine kit (R&D Systems) according to the manufacturer’s instructions. The amounts of cytochrome c in both the mitochondrial and cytoplasmic fractions were used to determine the ratio of cytoplasmic fractionation. The expression of Bcl family proteins was measured by ELISA using anti-Bak antibody (G-23), anti-Bax antibody (N-20), BCL-XL antibody (7D9), and anti-Bcl-xL antibody (H-5) (Santa Cruz Biotechnology) as primary antibodies, and biotinylated goat-anti-rabbit IgG as the secondary antibody with avidin-HRP (Boehringer).

### Semi-quantitative analysis of mRNA expression

Gene expression was determined by RT-PCR using the following primers: 5′-tacagcagcagcacaagatta-3′ and 5′-cgtgttctttccttctcagt-3′ for Sox6, 5′-tgccgcagcttctctgagcc-3′ and 5′-gctctgccgaggagatcacc-3′ for BMP-4, 5′-cctcattcacttacaccagtgagac-3′ and 5′-cagagccttcatacttcatacaccc-3′ for BMP receptor IA (BMPRIA), 5′-taacatgctcttacgaagctctggaa-3′ and 5′-gagctctgagactgctcgatcaagtc-3′ for BMP receptor IB (BMPRIB), 5′-atctctcatgaaaatgggac-3′ and 5′-tttccggtctcctgtcaac-3′ for BMP receptor II (BMPRII), and 5′-tgaaggtcggtgtgaacggatttggc-3′ and 5′-catgtaggccatgaggtccaccac-3′ for GAPDH, used as an internal control. RT reactions were performed using MuLV reverse transcriptase (ABI) and PCR was performed using KOD (Takara) according to the manufacturer’s instructions. To normalize for sample loading, the ratio of quantitative detection of each BMP-4 band to the corresponding G3PDH band was used.

### Quantitative analysis of BMP-4 by ELISA

Levels of intercellular BMP-4 and that in conditioned medium were determined by ELISA using mouse anti-human BMP-4 (R&D) as the primary antibody, with biotinylated sheep anti-mouse IgG (Amersham) and avidin-HRP (Boehringer) utilized as secondary antibodies.

### Neutralization of BMP-4 by anti-BMP-4 and anti-BMPR antibodies

P19 cells were cultured in bacterial-grade dishes in the presence or absence of RA and 50 ng/mL anti-BMP-4 antibody or 20 ng/mL anti-BMPR (BMPRIA, IB, or II) antibody for 48 h. Next, the cells were collected and suspended, and then stained with Hoechst 33342 or PI without fixation.

### Statistical analysis

Results are presented as mean ± SD values. Comparisons between multiple groups were performed using one-way ANOVA followed by Bonferroni/Dunn test. Differences were considered to be significant at *P* < 0.05.

## Results

### Sox6 suppression induced RA-dependent cell death in P19 cells

**Fig. 1:**
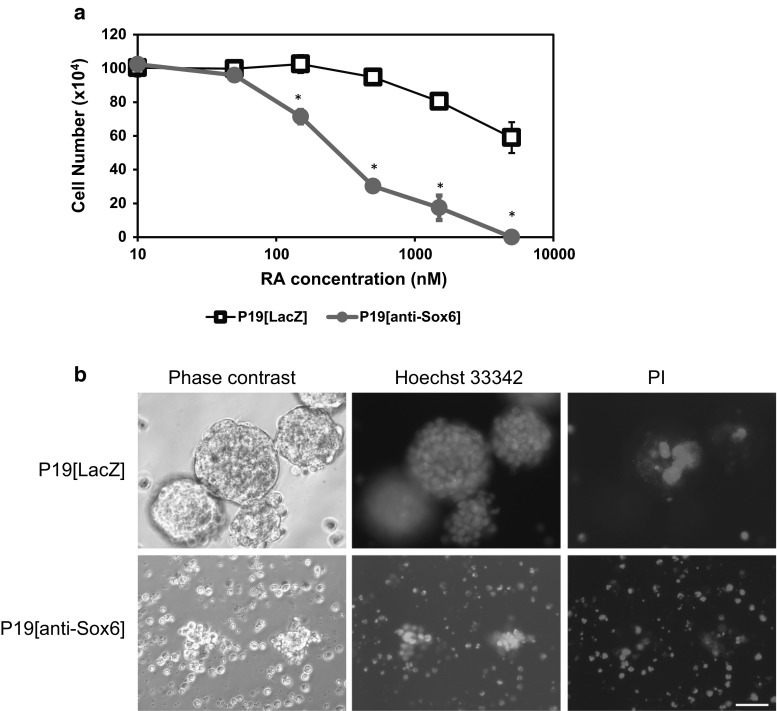
Sox6 suppression induced cell death in an RA dose-dependent manner. **a** P19[LacZ] and P19[Sox6] cells were cultured with various concentrations of RA for 48 h, then stained with trypan blue without fixation. *Bars* represent the mean ± SEM of three experiments in each group. **b** Cells were cultured with 500 nM RA for 48 h, then stained with Hoechst 33342 and PI for 30 min. *Scale bar* indicates 100 μm. *Asterisk* indicates differences that were considered to be significant at *P* < 0.05 by Scheffe’s *F* test

We previously reported that Sox6 suppression induced remarkable levels of P19 cell death during neuronal differentiation in the presence of RA [15]. In the present study, to examine the molecular mechanism underlying the cell death caused by Sox6 suppression, we first checked whether the Sox6 gene was expressed in P19[anti-Sox6] and P19[LacZ] cells, as control. Although RA induced cell death in a dose-dependent manner in both P19[anti-Sox6] and P19[LacZ] cells, the LD50 value of the P19[anti-Sox6] cells was 200 nM, remarkably lower than that of the P19[LacZ] cells at 5000 nM (Fig. 1a). Figure 1 shows phase contrast and fluorescence microphotograph images of P19[LacZ] and P19[anti-Sox6] cells at 4 days after addition of 500 nM RA, the usual condition for neuronal induction. P19[anti-Sox6] cells failed to form aggregates and showed large amounts of cell fragmentation and PI-stained dead cells (Fig. 1b, lower panels).

### Sox6 suppression induced activation of caspase 3 followed by caspase 9, but not caspase 8

**Fig. 2:**
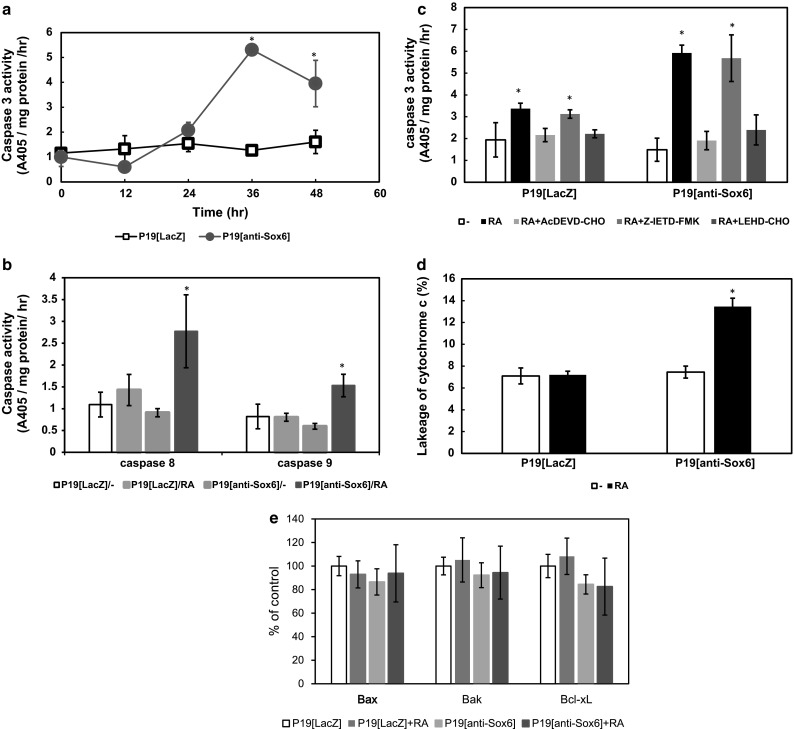
Sox6 suppression induced caspase 3 and 9 activation and leakage of cytochrome c. **a** Cells were incubated with 500 nM RA for 0–48 h, after which caspase 3-like activity was measured for 2 h using Ac-DEVD-pNA as the substrate. **b** Cells were cultured in bacterial-grade dishes with or without 500 nM RA for 36 h. Caspase 8- and 9-like activities were separately measured for 2 h using Ac-IETD-pNA as the substrate for caspase 8 and Ac-LEHD-pNA for caspase 9. **c** Cells were cultured in bacterial-grade dishes with or without 500 nM RA and the caspase inhibitors for 36 h, after which caspase 3-like activity was measured for 2 h using Ac-DEVD-pNA as the substrate. **d** Cells were cultured in bacterial-grade dishes with or without 500 nM RA for 36 h. Cytochrome c was measured by ELISA after subcellular fractionation. **e** Cells were cultured in bacterial-grade dishes with or without 500 nM RA for 36 h. The cytoplasmic Bcl-2 families were assessed by ELISA after subcellular fractionation. *Asterisk* indicates differences that were considered to be significant at *P* < 0.05 by Scheffe’s *F* test

To determine whether Sox6 suppression activates the caspase pathway in RA-treated P19 cells, we first measured caspase 3 activity levels in P19[anti-Sox6] and P19[LacZ] cells. Caspase 3 activity increased from 24 h after the addition of RA, and reached a peak at 36 h (Fig. 2a). Next, caspase 8 and 9 activities were measured at 36 h after addition of RA in P19[anti-Sox6] cells. Both were remarkably increased in P19[anti-Sox6] cells, whereas there was only a small increase in P19[LacZ]cells (Fig. 2b). To examine whether the activation of caspase 3 in P19[anti-Sox6] cells was mediated by an elevation in caspase 8 or 9 activity, we measured caspase 3 activity in P19[anti-Sox6] cells after incubation with RA and a separately added caspase 3, 8, or 9 inhibitor for 36 h. Caspase 3 activity was decreased by both the caspase 3 and 9 inhibitors, but not by the caspase 8 inhibitor (Fig. 2c). These results suggest that Sox6 suppression induces activation of caspase 3, followed by that of caspase 9.

We also measured cytochrome c levels in cytoplasmic fractions by ELISA. In P19[anti-Sox6] cells, a small amount of cytochrome c was detected in the cytoplasm after RA addition, whereas no such change was noted in the control cells (Fig. 2d). The cytoplasmic fractions of Bax, Bak, and Bcl-xL were determined to be factors related to this leakage of cytochrome c, but no changes were noted. These results indicate that Sox6 suppression causes leakage of cytochrome c from mitochondria, followed by activation of caspase 9 and continuous activation of caspase 3, after which apoptosis finally occurs.

### Sox6 suppression induced expression of BMP-4 following RA addition

When P19 cells are induced by RA for neuronal differentiation, approximately 90 % differentiate into neuronal cells, while the remainder undergo apoptosis. Gao et al. reported that the expression levels of BMP-4 increased during neuronal differentiation in RA-treated P19 cells [16], while another study found that addition of BMP-4 increased apoptosis by approximately fourfold in comparison with RA in P19 cells [17]. Therefore, we examined whether Sox6 suppression induces the expression of BMP-4.

**Fig. 3:**
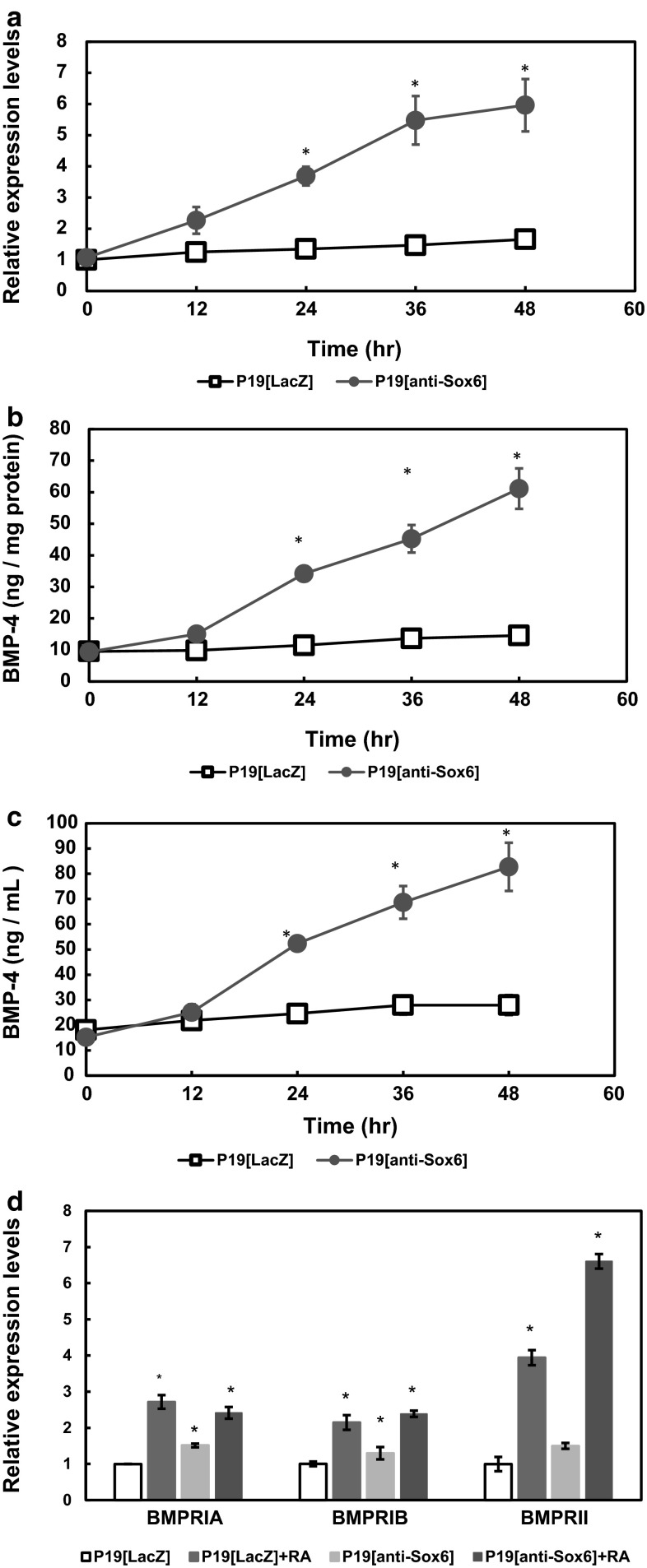
Sox6 suppression induced transcription, translation, and secretion of BMP-4, but not BMPRs. Cells were cultured in bacterial-grade dishes with or without 500 nM RA for 0–48 h, and then the mRNA expressions of **a** BMP-4 and **d** BMPRs were analyzed by RT-PCR. **a** To normalize for sample loading, the ratio of the value obtained in semi-quantitative determination of each BMP-4 band to the corresponding G3PDH band was used. BMP-4 protein levels were measured by ELISA using **b** cell lysates **c** and conditioned medium. *Asterisk* indicates differences that were considered to be significant at *P* < 0.05 by Scheffe’s *F* test

The time course of BMP-4 mRNA expression in RA-treated P19[anti-Sox6] cells is shown in Fig. 3a. The expression increased in a time-dependent manner following addition of RA in P19[anti-Sox6] cells, while it was not recognized in P19[LacZ] cells. Both intracellular and extracellular BMP-4 protein levels were significantly elevated in P19[Sox6] cells from 12 to 48 h after the addition of RA, while there was nearly no change in P19[LacZ] cells (Fig. 3b, c).

BMPs transduce signals by binding to heteromeric complexes of type 1 and type 2 serine–threonine kinase receptors. This binding to receptor complexes results in phosphorylation of intracellular Smads, which then translocate to the nuclei, where they regulate transcription [18]. The differential affinities of distinct BMP ligands for the three different type I receptors, BMP receptor type IA, IB, and II (BMPRIA, BMPRIB, and BMPRII, respectively), are thought to contribute to a diversity of actions during development [19, 20].

We examined the mediation of Sox6 suppression-induced cell death by the BMP receptors. Although BMPRII mRNA expression was most increased by Sox6 suppression and RA, each showed increases in both P19[anti-Sox6] and P19[LacZ] cells (Fig. 3d).

### Effects of BMP-4 and anti-BMP-4 antibody on cell death of P19[anti-Sox6] cells

**Fig. 4:**
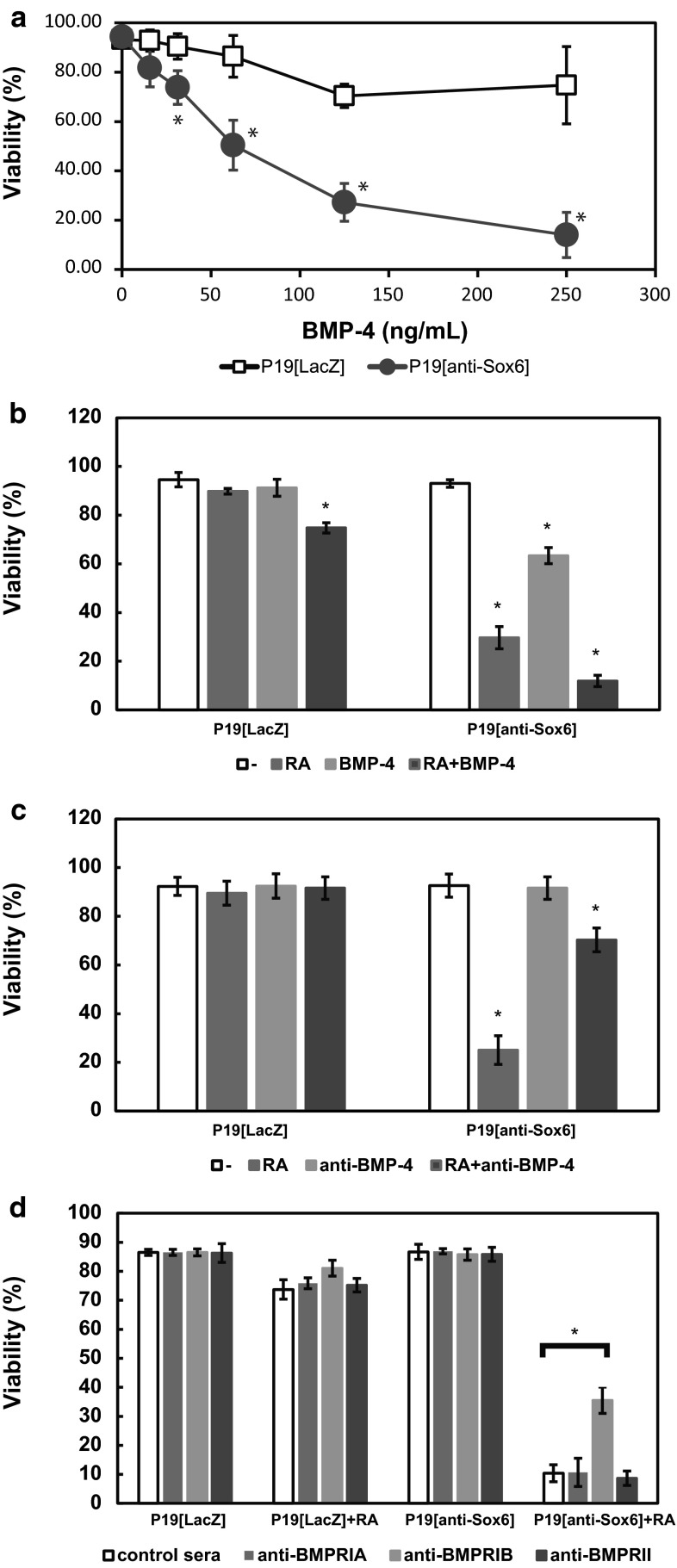
Apoptosis induced by Sox6 suppression may be mediated via BMP-4 and BMPRIB. **a** P19[LacZ] and P19[Sox6] cells were cultured with various concentrations of BMP-4 for 48 h, then stained with Hoechst 33342 and PI for 30 min. Cells were cultured for 48 h in bacterial-grade dishes with or without 500 nM RA and **b** 50 ng/mL of BMP-4, **c** 100 μg/mL of the anti-BMP antibody, or **d** the anti-BMPR antibody. The cells were then stained with Hoechst 33342 and PI without fixation

Since Sox6 suppression induced both production and secretion of BMP-4 following addition of RA (Fig. 3b, c), we examined the effects of added BMP-4 in those cells. P19[anti-Sox6] cells were cultured with various concentrations of BMP-4 for 48 h and the survival ratios were measured. Although BMP-4 induced cell death in a dose-dependent manner in both P19[anti-Sox6] cells and the LD50 value of the P19[anti-Sox6] cells was about 65 ng/mL, BMP-4 only caused a slight decrease in the survival ratio for P19[LacZ] cells (Fig. 4a). In P19[anti-Sox6] cells, RA induced about 80-90 ng/mL BMP-4 secretion (Fig. 3c) and 70 % cell death (Fig. 1a), and addition of BMP-4 without RA approximately correlated to these (Fig. 4a). When 50 ng/mL of BMP-4 was added to P19[anti-Sox6] cells, the survival ratio was reduced by approximately 70 % (Fig. 4b). In addition, in the presence of 500 nM RA and 50 ng/mL BMP-4, viability was remarkably decreased to about 10 % of that in the control cells.

We also examined the effects of the anti-BMP-4 antibody on P19[anti-Sox6] cell death. Cell viability after incubation with 500 nM RA decreased to about 25 % of that of the control, while incubation with the anti-BMP-4 antibody recovered viability to about 75 % of the control (Fig. 4c). Without RA, there was no change in viability by addition of the anti-BMP-4 antibody in both P19[LacZ] and P19[anti-Sox6] cells. Furthermore, antibodies for the 3 BMPRs were added to examine BMPR-mediated signal transduction related to cell death. Only the anti-BMPRIB antibody showed an effect, as approximately 30 % of the P19[anti-Sox6] cells recovered from cell death with its addition (Fig. 4d).

These findings suggest that Sox6 suppression induces transcription of BMP-4 and increases secretion of BMP-4. In addition, BMP-4 signaling is mediated by BMPRIB, with the result being apoptosis through the mitochondrial pathway.

## Discussion

Neuronal precursor cells occasionally malfunction during the neuronal differentiation stage, and such cell has been taken more selectively than a cell death [21]. We previously reported that Sox6 suppression caused apoptosis in the presence of RA [18], while the present study indicated that this effect occurs in an RA dose-dependent manner (Fig. 1). We also found that the survival ratio of P19[anti-Sox6] cells was about 25 % with the addition of RA at 500 nM, which is the level that induces neuronal differentiation of P19 cells. Sox6 enhances neuronal differentiation and is expressed in the induction stage of P19 cells with RA [11]. However, in the absence of Sox6, several different molecules related to apoptosis or survival may lose control.

Changes in caspase activity were examined in relation to molecule-related apoptosis, and we found that Sox6 suppression induced activation of caspase 3 in the presence of RA (Fig. 2). Moreover, activation of caspase 8 and 9, which are upstream of caspase 3, occurred. Since caspase 3 activity decreased when the cells were cultured with the caspase 9 inhibitor but not with the caspase 8 inhibitor, it is suggested that caspase 3 activity is increased by caspase 9 activation.

The apoptosis pathway mediated by caspase 9 is known to be a mitochondrial pathway [22]. We observed a small amount of cytochrome c leakage from mitochondria, though the expression levels of some Bcl-2 family members, such as Bax and Bak, promoters of apoptosis, and Bcl-xL, an apoptosis inhibitor, were not changed in the cytoplasmic fraction.

We also examined the signal molecule BMP-4 with regard to its involvement in Sox6 suppression through the mitochondrial pathway. BMP family members are growth factors that belong to the TGF-β superfamily and were originally identified as factors related to bone formation. Several reports have shown that these BMP members are deeply involved in morphogenesis, with BMP-2 and BMP-4 notable for their mesoderm-inducing activity, and are known to inhibit ectoderm development during embryogenesis [23–26]. In addition, it was reported that BMP-4 not only inhibits ectoderm differentiation, but also induces apoptosis of some neuronal precursors [27].

Glozak found that the number of cells that underwent apoptosis increased by 40 % when BMP-4 was added to wild-type P19 cells [18]. Thus, we examined whether Sox6 suppression induced the expression of BMP-4 and observed that BMP-4 mRNA was expressed, translated to protein, and secreted to the extracellular environment in the presence of RA (Fig. 3). BMP-4 induced cell death in a dose-dependent manner in P19[anti-Sox6] cells without RA (Fig. 4). However, even 250 ng/mL of BMP did not induce cell death in P19[LacZ] cells; thus, undifferentiated P19 cells have some degree of resistance against BMP-4, but the resistance is lost completely because of defects in Sox6. The addition of 50 ng/mL of BMP-4 caused apoptosis of P19[anti-Sox6] but not P19[LacZ] cells, while the addition of anti-BMP-4 antibody partially blocked cell death (Fig. 4). These results suggest that Sox6 suppression induces BMP-4, which mediates apoptosis through the mitochondrial pathway. Moreover, approximately 90 % of P19[anti-Sox6] cells treated with 500 nM RA and 50 ng/mL BMP-4 showed cell death, whereas only about 25 % of the P19[LacZ] cells showed cell death under the same conditions (Fig. 3).

The difference in sensitivity to BMP-4 may be related to BMPRs. At least three kinds of receptors have been reported, the kinase of the serine/threonine type, type 1 [BMPRIA (Alk 3), BMPRIB (Alk6)], and type II [28], which mediate the mesoderm induction and differentiation activities of BMP-4 [29]. The relationship among these receptors with regard to cell death is an interesting issue. Tank et al. reported that BMP-4 induced precocious interdigital cell death through BMPRIB. In our experiments, the expression levels of BMPRIA, IB, and II were increased by RA in both P19[LacZ] and P19[anti-Sox6] cells (Fig. 3). However, the anti-BMPRIB antibody only partially blocked cell death induced by Sox6 suppression and BMP-4 signaling was mediated by BMPRIB in P19[anti-Sox6] cells.

Sox6 is a transcriptional factor related to development and differentiation. The complex of Sox6, Sox5, and Sox9 binds to the Col2a1 gene encoding collagen-2 and activates transcription [30], while the homodimer of Sox6 functions as a suppressor of transcription [7]. Therefore, we speculate that RA induces transcriptional activation of BMP-4, whereas Sox6 suppresses BMP-4 expression, leading to survival of wild-type P19 cells.

We previously reported that Sox6 overexpression increased the expression of genes related to various types of neuronal differentiation and induced differentiation of P19 cells into a neuronal lineage without RA. Furthermore, suppression of Sox6 blocked neuronal differentiation and also dramatically induced RA-dependent cell death. These findings suggest that Sox6 plays a role in determining whether P19 cells differentiate to neuronal cells or undergo apoptosis. Our results showed that Sox6 suppression induced an increase in BMP-4 signals transduced via BMPRIB, which followed the mitochondrial pathway and finally activated the caspase cascade.

Apoptosis-mediated removal of cells that fail to differentiate is an important event. It might support that the mechanism is programed. Our findings may be helpful to explain the molecular mechanism of selective cell death. Normally, high expression levels of Sox6 lead to neuronal differentiation by RA in P19 cells, a kind of neuronal precursor cell. However, if Sox6 expression fails for some reason, Sox6 deficiency induces production and secretion of BMP-4, which mediates selective cell death. Therefore, the present results suggest that Sox6 protects from cell death by suppression of BMP-4 transcription.
